# Comparison of the In Vitro and In Vivo Electrochemical Performance of Bionic Electrodes

**DOI:** 10.3390/mi13010103

**Published:** 2022-01-09

**Authors:** Alexander R. Harris, Carrie Newbold, Dimitra Stathopoulos, Paul Carter, Robert Cowan, Gordon G. Wallace

**Affiliations:** 1 Aikenhead Centre for Medical Discovery, ARC Centre of Excellence for Electromaterials Science, Faculty of Medicine, Dentistry and Health Sciences, University of Melbourne, Melbourne 3010, Australia; 2 Department of Otolaryngology, University of Melbourne, 32 Gisborne Street, Melbourne 3002, Australia; carrie@mobibod.com (C.N.); dimitra@unimelb.edu.au (D.S.); 3 Cochlear Ltd., 1 University Avenue, Sydney 2109, Australia; pcarter@cochlear.com; 4 Department of Audiology & Speech Pathology, University of Melbourne, 550 Swanston Street, Melbourne 3010, Australia; r.cowan@unimelb.edu.au; 5 ARC Centre of Excellence for Electromaterials Science, Intelligent Polymer Research Institute, University of Wollongong, Wollongong 2522, Australia; gwallace@uow.edu.au

**Keywords:** electrochemistry, electrochemical impedance spectroscopy, animal model, bionics, electrode, testing protocol

## Abstract

The electrochemical performance of platinum electrodes was assessed in vitro and in vivo to determine the impact of electrode implantation and the relevance of in vitro testing in predicting in vivo behaviour. A significant change in electrochemical response was seen after electrode polarisation. As a result, initial in vitro measurements were poor predictors of subsequent measurements performed in vitro or in vivo. Charge storage capacity and charge density measurements from initial voltammetric measurements were not correlated with subsequent measurements. Electrode implantation also affected the electrochemical impedance. The typically reported impedance at 1 kHz was a very poor predictor of electrode performance. Lower frequencies were significantly more dependent on electrode properties, while higher frequencies were dependent on solution properties. Stronger correlations in impedance at low frequencies were seen between in vitro and in vivo measurements after electrode activation had occurred. Implanting the electrode increased the resistance of the electrochemical circuit, with bone having a higher resistivity than soft tissue. In contrast, protein fouling and fibrous tissue formation had a minimal impact on electrochemical response. In vivo electrochemical measurements also typically use a quasi-reference electrode, may operate in a 2-electrode system, and suffer from uncompensated resistance. The impact of these experimental conditions on electrochemical performance and the relevance of in vitro electrode assessment is discussed. Recommended in vitro testing protocols for assessing bionic electrodes are presented.

## 1. Introduction

Implantable electrodes can monitor and control the behaviour of excitable tissue, such as neurons and muscle fibres. These electrodes are used in research applications and increasingly in clinical devices to detect and control patient symptoms. One recent device involves placing electrodes on the surface of the brain to detect seizures in epileptic patients, allowing them to take steps to prevent injury [1]. A closed loop system may then be used to stimulate the affected tissue, preventing the seizure from occurring. Deep brain stimulation is being used to control symptoms associated with Parkinson’s disease, and is being trialled for other neurological disorders including depression [2]. Electrodes are also used to interface with the peripheral nervous system [3]. Cochlear implants are placed into the cochleae of patients with profound hearing loss [4]. Cochlear implants have developed significantly since their initial design, which assisted with lip reading, and now provide sufficient auditory cues enabling communication over the phone [5].

Despite the significant benefits of implantable electrodes, cochlear implants still do not provide natural sensory input, with some recipients having poor sound perception in the presence of background noise and a poor appreciation of music [6,7]; deep brain stimulation only controls patient symptoms, but does not prevent disease progression [8]; and our understanding of what qualifies as an epileptic seizure as opposed to normal neural function is poor, impacting how these types of devices can be used clinically [9].

The implantation of electrodes is highly invasive, and subsequently they are expected to function correctly for the remainder of the patient’s lifetime. However, electrode corrosion, device breakage, battery failure, and encapsulation by scar tissue are some of the issues that may impact device performance after implantation [10,11]. Improved device performance may be achieved by altering the electrode design or materials. For instance, this may allow a greater control of current to the target tissue, preventing off target side effects, the prevention of electrode corrosion, or reduced power usage. The development and modification of a clinical device is a costly and time-consuming activity, so there is a great incentive to ensure any proposed designs have a strong likelihood of success.

Bionic implants are electrochemical devices, converting electrical current into ionic current at the electrode–tissue interface [12]. Electrochemical processes occur at this interface, and an understanding of these mechanisms is required to ensure safe and effective device function. Various in vitro electrochemical measurements are used to characterise these electrodes in the hope that they accurately reflect their in vivo performance [12,13]. Changes in electrochemical response after implantation are then typically attributed to catastrophic failures of the electrode or fibrous tissue formation around the implant with limited experimental evidence [14]. These electrochemical responses are often further simplified to a single charge storage capacity value or impedance at 1 kHz, with the aim of an easy comparison of electrode function over time or between electrodes [15,16,17,18,19], but which ignores the electrochemical mechanisms occurring at the electrode–tissue interface. This may be due to the incorrect assumption that the electrode–tissue interface acts as pure electrical circuit and not an electrochemical system. It is also common for implantable electrodes to be classified as either a capacitative or Faradaic device, so that a charge passed across the electrode–tissue interface is only due the movement of ions or electrons [20]. In reality, no electrode is purely capacitative or Faradaic, and numerous experimental conditions affect the electrochemical behaviour of this interface [21]. As a result, certain experimental conditions used for in vitro testing may have very poor correlation to the in vivo environment and give incorrect information about an electrode’s performance.

To understand the relevance of in vitro electrochemical testing, this article measures the electrochemical performance of platinum cochlear implants over time in vitro and in vivo. The impact of electrode aging, implantation, protein fouling, fibrous tissue formation, and electrode polarisation are assessed. The implication of electrode activation and implantation on the relevance of in vitro testing is then discussed. Finally, the most relevant in vitro testing protocol for assessing bionic electrodes is presented.

## 2. Methods

### 2.1. Materials

Twenty cochlear implant (CI) electrode arrays, each containing four full band platinum electrodes (*n* = 80), were fabricated in house. The electrode array tapered from 0.47 mm to 0.41 mm in diameter. Electrode rings were 0.3 mm wide with 0.45 mm spacing and embedded in a silicone rubber carrier. This created electrodes with geometric areas ranging from approximately 0.39 mm^2^ to 0.44 mm^2^. Array lead wires were joined to silicone-coated stainless-steel wires ending in stainless steel contacts, which were covered by a silicone rubber cap after the final measurement before wound closure in vivo or electrodes were sealed in saline solution for aging studies. The electrodes were sterilised in a steam autoclave before use, but no mechanical polishing or electrochemical cleaning was performed.

Electrodes were tested in vitro in a 3-electrode configuration with a Ag/AgCl (3 M KCl) reference and Pt wire counter electrode. The in vitro testing solution was sterile sodium chloride (Promedica, Melbourne). No degassing was performed. A SI1287 potentiostat and SI1260 impedance analyser (Solartron, Leicester) were used to perform cyclic voltammetry and electrochemical impedance spectroscopy (EIS). EIS was undertaken at 0 V with a 10 mV amplitude over a frequency range of 0.1–100,000 Hz. A single cyclic voltammogram was then performed, starting and ending at 0 V, scanning in a cathodic direction initially, with maximum and minimum potentials of 0.8 and −0.8 V at a scan rate of 20 mV s^−1^. Equivalent circuit fitting of the EIS data was performed with ZView (Scribner, North Carolina).

### 2.2. Surgical Procedures

Six Dunkin–Hartley tri-colour guinea pigs were used to test 3 electrode arrays each, one in each cochlea and a third subcutaneously (18 electrode arrays total). All experimental procedures were approved by the Bionics Institute Animal Research & Ethics Committee (16/356AU). Prior to surgery, animals were anaesthetised with isoflurane (2%, oxygen flow 1 L/min). Body temperature was regulated with a heated surgical table. Fur was removed from behind both ears and the back. Local anaesthetic (2% lignocaine, subcutaneous), analgesic (0.03 mg/kg buprenorphine, subcutaneous), and atropine sulphate (0.06 mg/kg, intramuscular) were given. An incision was made to expose the mastoid bulla and a burr hole drilled through the bulla to expose the round window and lower hook turn. A cochleostomy was performed into the scala tympani of the basal turn. The CI was inserted, with all four electrodes placed in the cochlea in all bar one animal, where only 3 electrodes fit inside the cochlea. Both cochleae of each animal were implanted before an incision was made ~4 cm lateral to the spine for implanting a subcutaneous electrode. Two platinum wires, used for quasi-reference and counter electrodes, were placed in the extracochlear tissue or adjacent to the subcutaneous electrode prior to electrochemical testing and removed after use.

Electrodes were initially tested electrochemically in saline, implanted and tested electrochemically in vivo (implanted time period of ~120 min), then removed and gently rinsed in saline to remove any loosely attached tissue before retesting in saline to assess the effect of acute implantation. The exposed electrical contacts were then sealed in a silicone tube and the arrays reimplanted into their initial cochlea or subcutaneous pocket. The distal portion of the lead wire was wedged into the bullostomy and superglue applied (dental cement was not used to fix the CI so that it could be removed at the conclusion of the testing period without destroying the cochlea). The wound was closed by internal suture of the muscle and stapled (Leukoclip SD staples). All animals recovered from anaesthesia and were given a broad-spectrum antibiotic (5 mg/kg enrofloxacin, subcutaneous) and supplemental fluid replacement (Hartmann’s solution). The total time for the electrode testing and surgery of each animal was ~5 h.

For final electrode testing, animals were anaesthetised (ketamine 60 mg/kg, ilium xylazil 4 mg/kg in a 3:1 ratio) and the electrodes re-exposed. The silicone tube on the electrical contacts was removed by applying chloroform. Platinum wire quasi-reference and counter electrodes were reimplanted and the electrodes retested electrochemically. Care was taken to avoid moving the electrode arrays from within any encapsulating scar tissue. After testing, the electrode arrays were removed from the animal, gently rinsed in saline, and tested again in fresh saline. To assess the impact of tissue encapsulation over time, animals were retested at different intervals, one at 4 days, one at 11 days, one at 14 days, one at 48 days, and two at 8 weeks.

At the conclusion of each experiment, animals were deeply anaesthetised (Lethabarb, Virbac Animal Health, Australia, 2.5 mL, intraperitoneal) and perfused transcardially with heparin 1 mL/L in saline at 37 °C and then 10% neutral buffered formalin at 4 °C. Representative cochleae were harvested at 48 days and 8 weeks. The bullae were placed in the formalin for a further 24 h and then decalcified in 10% ethylenediaminetetraacetic acid in phosphate buffered saline over approximately 14 days. The bullae were embedded in Tissue Tek Optimal cutting temperature compound and oriented for histological processing. Cochleae were sectioned on a mid-modiolar plane at intervals of 12 µm using a cryostat. Every third section was stained with haematoxylin and eosin (H&E) stains and the area of the scala tympani occupied by loose or dense fibrous tissue or new bone was assessed from a light field image (Zeiss Axioplan 2).

A control electrode array was also tested initially in saline. The electrical contacts were sealed in a silicone tube and the array placed in sterilised PBS. After 2 weeks the array was removed from solution, rinsed in deionised water, and the silicone tube was removed and retested in saline. The silicon tube was then reapplied and the array placed back into resterilised PBS. The electrode array was again retested after 8 weeks. A second control electrode was tested multiple times in succession in saline without storing.

## 3. Results

### 3.1. Voltammetric Response in Saline

All electrodes were initially tested in non-degassed saline. Electrochemical impedance spectroscopy was performed initially, followed by cyclic voltammetry. This provided an EIS response before any electrode activation processes occurred, due to electrode polarisation. EIS was performed at 0 V and with a 10 mV sinusoidal amplitude, resulting in negligible electrode polarisation. No current pulsing was performed in this study to further prevent any electrode polarization effects that would limit data analysis.

**Figure 1:**
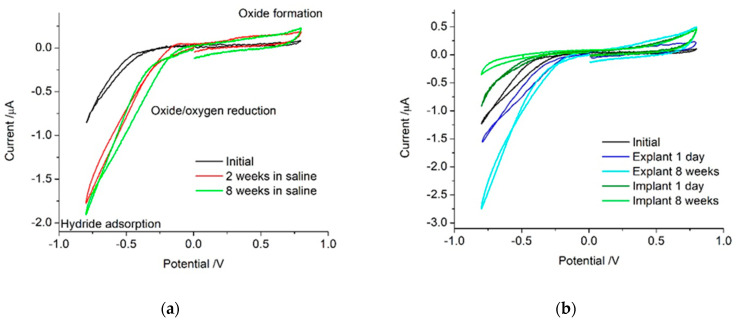
Voltammetric cycle of a platinum cochlear implant electrode from 0 → −0.8 → 0.8 → 0 V at a scan rate of 20 mV s^−1^: (**a**) electrode tested and stored in saline over 8 weeks and (**b**) electrode initially tested in saline, implanted in a cochlea for 1 day, explanted after 1 day, reimplanted in cochlea for 8 weeks, and explanted after 8 weeks.

Cyclic voltammetry was performed from 0 V to −0.8, up to 0.8 and back to 0 V vs. Ag/AgCl (3M KCl) at a scan rate of 20 mV s^−1^ (Figure 1). This potential range was chosen as previous work showed water oxidation and reduction proceeded at higher voltages [22]. The scan was started and finished at 0 V to minimise any polarisation effects that may occur from starting and finishing at 0.8 V. A slow scan rate was used to minimise any uncompensated resistance (*iR*_u_) effects when the electrodes were implanted. At higher scan rates, the current magnitude increases, and high resistances can lead to a distortion of the voltammetric response.

The voltammetric response will be described initially, as it provides details on the electrochemical mechanisms occurring at the platinum electrode. On the reduction sweep, a large current occurred at potentials below −0.5 V (Figure 1a). This is mainly due to oxygen reduction [23], and at lower potentials, hydrogen adsorption. On switching the scan direction, a small capacitance current was present, but no oxidation process was visible.

On subsequent cycles, either performed immediately (data not shown) or after 2 and 8 weeks of storage in saline, the reduction process shifted to more positive potentials. On the positive sweep, a small, broad oxidation process occurred from around 200 mV, consistent with platinum oxide formation and anion adsorption [21].

Typically, the voltammetric response is converted into a potential-time plot and integrated to provide a charge value, which can then be divided by the electrode area to provide the charge storage capacity (CSC). CSC is related to the change in electrode potential which occurs when current pulses are applied to stimulate neural tissue [24]. When starting the voltammetric experiment in the middle of the potential range and only running one cycle, there is no complete, continuous reduction sweep obtained, preventing measurement of the cathodic CSC. The charge passed on the initial oxidation sweep in Figure 1 was 2.1 µC, increasing to 4.8 and 4.2 µC after 2 and 8 weeks of storage. The electrode area varies over the array, but with an assumed area of 0.4 mm^2^, an anodic CSC would initially be ~0.52 mC cm^−2^, increasing to ~1.2 mC cm^−2^ after 2 weeks of storage. This is within the lower end of the anodic CSC range typically seen for platinum [21]. The charge passed during voltammetry is due to several reaction mechanisms [13] but is dominated by oxygen reduction, and changes significantly with the number of voltammetric cycles. Subsequently, the CSC obtained from a new platinum electrode in vitro is a poor predictor of subsequent CSC measurements.

### 3.2. Impedance Response in Saline

**Figure 2:**
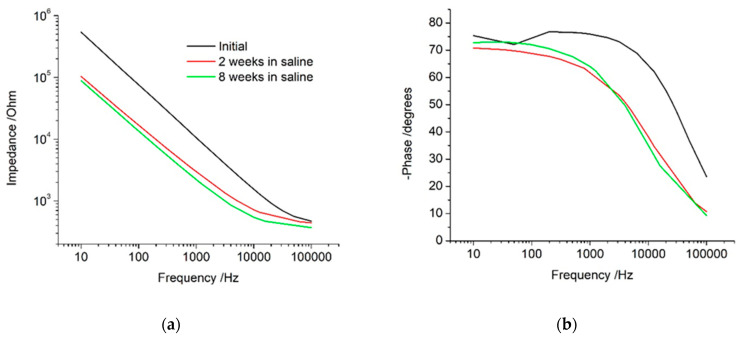
(**a**) Impedance; (**b**) Phase Angle. Electrochemical impedance spectroscopy of a platinum cochlear implant electrode at 0 V with an AC amplitude of 10 mV before cyclic voltammetry. Electrode tested and stored in saline over 8 weeks.

A new electrode placed in saline generates a near linear impedance response versus frequency (Figure 2a). The phase angle was around −20 degrees at high frequencies and approached −80 degrees at lower frequencies (Figure 2b). This indicates the electrochemical cell is dominated by resistance behaviour at high frequencies and capacitance at low frequencies. The impedance appears as a near linear response in the Nyquist plot, with a steep gradient (Figure S1). After cyclic voltammetry (either performed immediately or after storage in saline for 2 and 8 weeks), the impedance response decreased in magnitude. The transition of the phase angle from −20 to −80 degrees shifted to lower frequencies.

**Figure 3:**
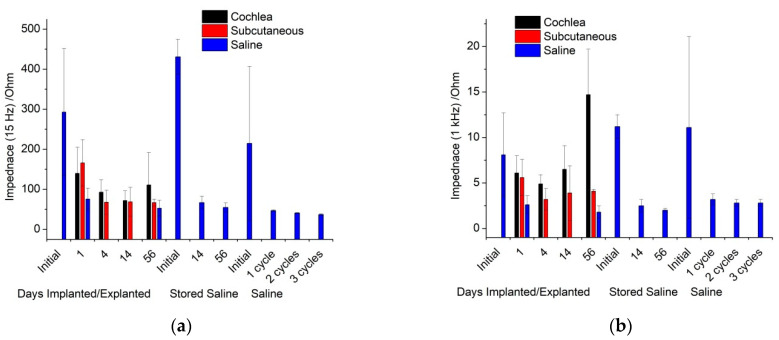
Impedance at (**a**) 15 Hz and (**b**) 1 kHz over time; initial, explanted, or stored electrode response in saline, or in vivo response in the cochlea or subcutaneously.

The impedance at 1 kHz is typically reported for bionic electrodes. An initial impedance of ~11 kΩ decreased to ~2–3 kΩ after voltammetry (Figure 3 and Table S1). The impedance at low frequencies has a greater relationship to the electrodes’ behaviour [25,26]; it also decreased significantly from an initial value of ~200–400 kΩ to ~30–70 kΩ, decreasing with successive voltammetric cycles.

**Figure 4:**
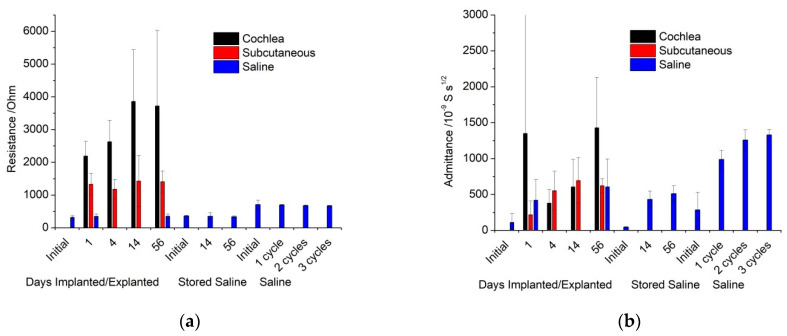
Equivalent circuit parameters (**a**) resistance and (**b**) admittance over time; initial, explanted, or stored electrode response in saline, or in vivo response in the cochlea or subcutaneously.

With EIS being performed at 0 V, there are no Faradaic reactions occurring. Therefore, EIS was fit with a simple equivalent circuit consisting of a resistor (R) in series with a constant phase element (CPE). The resistor accounts for the solution resistance, while the CPE models the electrode/electrolyte solution double layer interface. The use of a constant phase element rather than a capacitor is usually explained as inhomogeneous current distribution, typically due to surface roughness or inhomogeneity in the chemical functionality of the surface, including ion adsorption [27]. Fitting this equivalent circuit generates the parameters resistance (*R*), and an admittance (*Q*_0_) and power (*n*) term for the constant phase element (Figure 4 and Table S2). For all experiments performed in saline, *R* is almost constant. In contrast, *Q*_0_ increases significantly after one voltammetric cycle, while *n* may decrease slightly. The quality of the fit is displayed in Figure S1 and an average χ^2^ over all electrodes at all time points and conditions was 3.6 × 10^−4^.

### 3.3. Voltammetric Response In Vivo

Following initial electrochemical testing, electrodes were either implanted into the cochlea or subcutaneously. In vivo electrochemical testing was still performed in a 3-electrode setup, but with a quasi-platinum reference electrode. The distance between electrodes was less than one inch. Electrochemical testing began after all electrodes were implanted, with the total testing time being ~2 h. Once again, EIS was performed initially to minimise electrode polarisation effects. However, the voltammetric response will be described first.

The voltammetric behaviour of the platinum electrode after implantation is similar in shape to saline (Figure 1b). However, the current associated with the oxygen reduction process below −0.5 V decreased in magnitude and the oxidation current above 0.5 V was slightly larger. The same response was seen on the electrodes implanted in the cochlea or subcutaneously. When this electrode was explanted and retested in saline, there was an immediate increase in the reduction current below −0.5 V and decrease in the oxidation current above 0.5 V. The explanted electrode response was very similar to the control electrodes that were retested in saline immediately or after 2 weeks of storage in saline; the reduction and oxidation currents increased in magnitude with electrode activation. This implies these voltammetric changes, when performed in vivo, are due to the media surrounding the electrode/tissue interface and not changes to the electrode surface.

The electrodes were then reimplanted between 4 days to 8 weeks to allow a fibrotic response to occur around the electrode. After a period of time, the electrode contacts were re-exposed and the electrochemical response retested. An electrode implanted for 8 weeks displayed a further decrease in current magnitude below −0.5 V, but the oxidation current was unchanged. Again, there were no differences detected from the implantation site or length of implantation time. The electrodes were then explanted and retested in saline. There was a significant increase in reduction current below −0.25 V while the oxidation current remained similar in magnitude. The explanted electrode response was very similar to that of electrodes stored in saline for 8 weeks.

The initial anodic charge was 2.5 µC; when implanted, it increased to 8.1 µC, decreasing to 5.7 µC when explanted, then increasing again to 8.6 µC when implanted for 8 weeks and 8.8 µC after explanting (Figure 1b).

### 3.4. Impedance Response In Vivo

**Figure 5:**
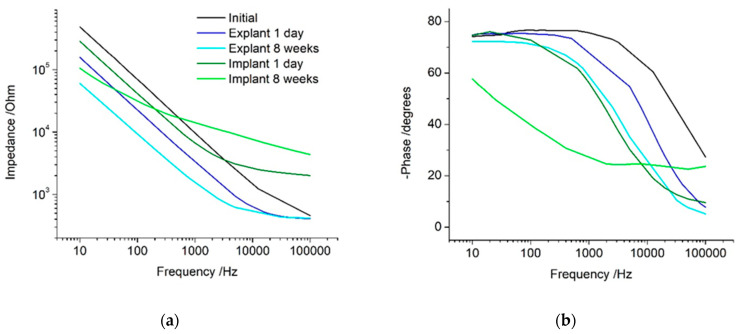
(**a**) Impedance; (**b**) Phase Angle. Electrochemical impedance spectroscopy of a platinum cochlear implant electrode at 0 V with an AC amplitude of 10 mV before cyclic voltammetry. Electrode initially tested in saline, implanted in a cochlea for 1 day, explanted after 1 day, reimplanted in cochlea for 8 weeks, and explanted after 8 weeks.

The impedance response recorded in vivo displayed a more constant value at high frequencies, rising in impedance at lower frequencies (Figure 5a). The phase angle was around –10 degrees at high frequencies, shifting to −75 degrees at lower frequencies (Figure 5b). When the electrode was explanted, the impedance at all frequencies decreased and the transition in the phase angle shifted to higher frequencies.

The electrode was then reimplanted for 8 weeks before retesting. After 8 weeks, the impedance at high frequencies increased further, while the impedance at low frequencies decreased. The phase angle remained at −25 degrees over a wide frequency range before rising towards −60 at lower frequencies. When the electrode was explanted again, the impedance decreased significantly over all frequencies. Compared to the initial explant response, the impedance at high frequencies was equivalent, while low frequencies were reduced. The phase angle also resembled the response after initial explant, with a slight shift to lower frequencies. The overall explanted impedance response after 8 weeks in vivo was very similar to an electrode that had been stored in saline for 8 weeks.

The impedance at 1 kHz was measured across time and implantation site (Figure 3 and Table S1). There was a slight decrease in impedance after the initial measurement. The response in the cochleae increased slightly over time and the explanted value was lower than the implanted response. The impedance was also measured at 15 Hz. There was a significant decrease in impedance after the initial measurement. The explanted value was slightly lower than implanted measurements, but there were no trends over the implantation period.

The implanted impedance response was fit with the same equivalent circuit (Figure 4 and Table S2). The solution resistance in saline was consistent across time and after explant from any in vivo site. In contrast, it increased significantly when measured in vivo. It was over 1 kΩ when the electrode was subcutaneous and over 2 kΩ when in the cochlea. The subcutaneous electrodes were relatively constant over 8 weeks of implantation, while those in the cochleae increased over time and were more variable. Consistent with the in vitro measurements, the admittance term increased significantly after the initial measurement, with some small variations over subsequent time. There may also be a slight decrease in the power term from the initial measurement.

### 3.5. Histology of Cochleae

Following electrode removal, cochleae were harvested and assessed for fibrotic response (Figure S2). Loose and dense fibrotic tissue and bone formation were seen in all cochleae tested after 48 days to 8 weeks of implantation (Table 1). The amount of fibrous tissue formed was consistent with previous work performed in our lab. At shorter time periods, the levels of bone formation are expected to be slightly lower. The electrode would also fill a significant volume of the scala tympani; however, it was removed prior to histological assessment, preventing its quantification. The amount of fibrous tissue formed around the subcutaneous electrodes could not be assessed after electrode removal.

## 4. Discussion

### 4.1. General Considerations of Electrochemical Mechanisms Occurring at an Electrode Surface In Vitro and In Vivo

Cyclic voltammetry allows the investigation of electrochemical reaction mechanisms occurring at an electrode surface. Platinum is able to support a range of Faradaic and pseudo-capacitance (surface confined Faradaic) reactions as well as capacitance [13]. In saline, this includes the reduction and oxidation of platinum oxide, adsorption and stripping of hydrogen, anion adsorption, reduction of oxygen, and reduction and oxidation of water. The mechanisms that do occur depend on the electrode surface, solution composition, and applied waveform [21]. The initial platinum cochlear implant electrode showed no electrochemically detectable levels of platinum oxide present (Figure 1). On its own, voltammetry is not able to distinguish between different mechanisms occurring under the broad reduction process below −0.5 V. In non-degassed saline on a sterilised but unpolished platinum cochlear implant electrode, the reactions that occurred within the water window were most likely oxygen reduction, small amounts of platinum oxide reduction and formation, anion adsorption, capacitance, and possibly hydrogen adsorption. After multiple potential cycles, the platinum is increasingly oxidised, increasing the current associated with platinum oxide formation and removal. Electrode activation processes also reduced the over-potential required for the platinum oxide and oxygen reduction processes, shifting them to more positive potentials.

When the electrode was implanted, the surrounding tissue contained a range of organic species that may be electrochemically active or affect the reactions detected in saline. The implanted electrode voltammetry showed a smaller reduction current magnitude below −0.5 V than seen on the explanted electrode (Figure 1). This suggests a lower oxygen tension in tissue than in the non-degassed saline. In the body, most oxygen is bound to haemoglobin, so the oxygen tension available for reduction at the electrode surface is expected to be extremely low [23,28,29]. The 1-day implanted electrode also had a larger oxidation current above 0.5 V than the explanted electrode. This may be due to the oxidation of organic molecules or anion adsorption from species not present in saline [21,30,31]. After 8 weeks, the implanted electrode had an even smaller current associated with oxygen and platinum oxide reduction. However, the oxidation process appeared similar to that of the 1-day implant. This implies the electrode is not being oxidised while it is implanted and the species responsible for the oxidation current is still present. The 8-week explanted electrode still generates a large oxygen reduction current, indicating the surface is highly active and not blocked by organic species.

When judging the utility of the in vitro voltammetric analysis of electrodes as a model for in vivo performance, the solution composition is of critical importance. Similar ionic concentration and composition, low oxygen tension, and similar pH and organic composition are required [21,32,33,34,35]. While saline or similar buffered electrolyte solutions offer reasonable, simple-to-use testing conditions, trace components in vivo may affect the voltammetric response. Furthermore, electrode polarisation can significantly modify its behaviour. Subsequently, voltammetry should be performed on implantable electrodes to gauge the range of reactions that can occur at the electrode–tissue interface. However, the simplification of this analysis to a charge storage capacity or charge density value may not provide any useful or relevant information on its in vivo performance.

### 4.2. Comparison of Impedance Response In Vitro and In Vivo

The impedance of bionic electrodes is typically measured with the value at 1 kHz reported. A lower electrode impedance is related to its thermal noise and increased signal-to-noise ratio for neural recording [34]. We recently showed that impedance at low frequencies, rather than at 1 kHz, is more strongly dependant on the electrode properties and is a better predictor of thermal noise and the signal-to-noise ratio of neural recordings [26]. These previous measurements were made on electrodes that were not electrically polarised, nor was the impedance measured in vivo. To further understand the impedance behaviour of bionic electrodes, in the current work, the impedance was measured before and after electrode polarisation and before and after implantation.

The platinum electrode behaved as a CPE in series with a resistance, in saline, when implanted in the cochlea or subcutaneously. Electrode polarisation and fibrous tissue formation had no effect on the equivalent circuit required to achieve a good fit of the impedance response. Importantly, this indicates that tissue and fibrous tissue do not introduce a new Warburg element or time constant into the impedance response.

Impedance was performed at 0 V, where voltammetry indicated minimal Faradaic charge transfer was present. As a result, no Warburg element was required in the equivalent circuit. However, if the impedance was performed below −0.5 V or above 0.5 V, a Faradaic current due to oxygen reduction or platinum oxidation would have been present. While these applied potentials during EIS are unlikely to be used, the presence of Faradaic reactions at the measurement potential may occur in different conditions or on other electrode materials. The impedance response would then have been dependant on the Faradaic reaction, including the concentration of the redox species. For instance, impedance performed at −0.5 V would have shown a significant effect at low frequencies due to oxygen. This would result in variations between the non-degassed in vitro response and the low oxygen tension in vivo response. Similarly, potentials above 0.5 V may be affected by a Faradaic current associated with anions and organic species. The magnitude of the Faradaic current at these potentials also depends on the electrode surface, with electrode activation increasing the current magnitude and subsequently affecting the impedance [21].

Great care must then be taken when performing and analysing impedance in vitro as a model of in vivo behaviour. Once again, the solution composition for an in vitro test must be similar to the in vivo environment. The choice of measurement potential and correct fitting of an equivalent circuit is crucial in correctly identifying the mechanisms occurring at the electrode–tissue interface.

Impedance performed in vitro displayed a reproducible solution resistance across all electrodes and measurement times (Table S2). Implanted electrodes had a larger resistance, with the cochlear electrodes double that of the subcutaneous electrodes. This is consistent with the current from the cochlear electrodes having to pass through more resistive bone. There was some variable increase in cochlear electrode resistance over time, which was most likely due to bone formation in the scala tympani. The consistency in resistance over time of the subcutaneous electrodes and some cochlear electrodes indicates that fibrous tissue does not have a significant difference in resistivity compared to normal tissue. The similarity of the initial and explanted electrode resistivity also indicates that any protein fouling has a minimal impact on impedance. The effects of protein fouling on electrodes in vitro were studied previously [35]. When an electrode was placed into a protein containing solution with a similar composition to perilymph, it partially blocked the electrode, resulting in impedance at 12 Hz increasing by ~20–30 kΩ and admittance decreasing slightly. Therefore, protein fouling is expected to be occurring on the implanted electrodes, but it has a significantly smaller effect than electrode activation and measuring within tissue (Tables S1 and S2).

Admittance increased significantly after electrode polarisation, but no trends were visible between in vitro, either cochlea or subcutaneous implants, or over time. The admittance value is a function of surface area, roughness, and chemical functionality. Similar increases in admittance have been seen on the oxidation of platinum and iridium Utah and Michigan style bionic electrodes [36,37,38]. Electrode polarisation drove changes to the electrode surface, but changes to the surrounding solution or tissue composition had a minimal impact on the admittance.

The impedance response is dominated by the electrode behaviour at low frequencies and by the solution properties at high frequencies. The transition between these two frequency regions is termed the Maxwell–Wagner frequency [26]. This frequency depends on testing conditions and is difficult to measure and predict. When impedance analysis is limited to a measurement at 1 kHz, it is seen to decrease under all conditions, with only small differences between the implanted and control electrodes. This gives the impression that the electrode is improving in behaviour when implanted and over time, and that the in vitro and in vivo responses are similar. This measurement did not resolve any changes due to electrode activation or increases in tissue resistivity. If a Faradaic current was present, then the 1 kHz measurement would also be affected by the electron transfer reaction. The measurement frequency appears to be near the Maxwell–Wagner frequency, and therefore is susceptible to changes in most experimental conditions, which cannot be resolved. Subsequently, articles that only report the impedance at this frequency do not provide sufficient information to assess the electrode or tissue behaviour and any analysis of the results must be viewed with caution.

The impedance at low frequencies was seen to decrease with electrode activation and admittance. This frequency was previously shown to strongly correlate with electrode area, thermal noise, and signal-to-noise ratio [26,35]. Reporting of in vitro impedance at low frequencies would therefore be a far better predictor of in vivo performance. The impedance at high frequencies is strongly correlated with the solution resistivity. It would therefore be a much better measure of tissue resistivity over time than impedance measurements at 1 kHz.

### 4.3. General Experimental Considerations for the Electrochemical Analysis of Bionic Electrodes

**Figure 6:**
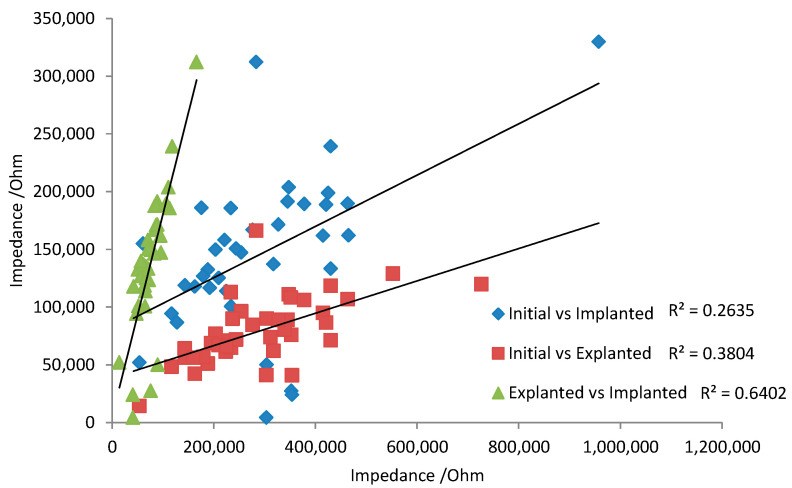
Correlation of impedance at 15 Hz. Initial saline measurement vs. implanted for 1 day, initial saline measurement vs. explanted after 1 day, and explanted vs. implanted for 1 day.

There are some general aspects that must be taken into account when translating the in vitro and in vivo electrochemical response. Over 8 weeks of implantation, there appeared to be little change in voltammetric response. This implies the tissue is not noticeably oxidising or damaging the electrode. For neural recording electrodes, the electrochemical response of platinum may therefore be relatively consistent, so that initial electrochemical measurements can be correlated with later in vivo performance. However, the electrodes were not electrically stimulated while implanted. Electrode polarisation during neural stimulation may induce electrode oxidation and surface rearrangement. Subsequently, the electrochemical behaviour of stimulated electrodes may change over time. Changes in electrode behaviour would reduce any correlation from initial electrochemical analysis with later in vivo performance. For instance, the impedance at 15 Hz was compared between initial, 1-day in vitro, and 1-day explanted responses (Figure 6). There was a very poor prediction of in vivo response with the initial measurement due to the activation of the electrode from one voltammetric cycle. A stronger correlation of 1-day in vivo and 1-day explanted impedance was achieved, as electrode polarisation from subsequent voltammetric cycles had less impact on the impedance response.

The electrochemistry performed in saline was with a defined and stable reference electrode. This allows the potentials of any processes to be measured accurately. The solution composition can also be carefully modified to assign voltammetric responses to specific reaction mechanisms. In contrast, the in vivo measurements were made versus a quasi-reference electrode. The potential of a quasi-reference electrode is unknown and can change over time with changes in tissue composition and electrode surface, even within a single experiment. For instance, oxygen concentration, pH, platinum oxide formation, anion concentration, and organic species adsorption can alter the reference electrode potential. The potential of a platinum electrode coated with varies proteins changed by nearly 100 mV [37]. It is possible to calibrate a quasi-reference electrode by measuring the potential of a known redox process. Typical redox species used for calibrating a quasi-reference electrode include ferrocene, ferricyanide and ruthenium hexaamine. When the concentration of these species is known, the oxidation and reduction peaks of their voltammetric response can be directly translated to the potential on a known reference electrode. However, the addition of these species into an animal may induce an undesirable biological response. Furthermore, the concentration of the chemical would not be accurately known. A well-defined surface confined redox process can also be used to calibrate the electrode potentials. The platinum oxide reduction process and hydrogen adsorption are surface confined; however, in opposition to previous reports [39], these processes are poorly defined and depend on solution composition (pH and anion concentration) that prevent their use as a calibration process. As a result, the voltammetric response obtained in vivo may not align with the measurements in saline. This will affect the potential range used for defining the safe water electrolysis window, assigning potentials to specific redox reactions, and obtaining equivalent charge and CSC values from in vitro and in vivo experiments. Great care must then be taken in assigning specific potentials and reaction mechanisms for redox processes from the in vivo electrochemistry.

EIS was performed at 0 V vs. the reference electrodes, with the in vivo quasi-reference electrode being different to the well-defined Ag/AgCl reference electrode. The actual working electrode potential was therefore different for the in vitro and in vivo experiments. No Faradaic process was present at this potential, so the equivalent circuit was valid for in vitro and in vivo measurements. Changes to electrode potential do not affect the solution resistance, but will change the admittance value by ~10–20% [35]; however, this is significantly smaller than the order of magnitude change seen from electrode activation (Table S2).

All electrochemical measurements performed in this manuscript were undertaken in a 3-electrode system. A 3-electrode system ensures the majority of the current flows from the working electrode to a counter electrode while the working electrode potential is controlled by the reference electrode. In a commonly used 2-electrode system, the current flows from the working electrode through the reference electrode [39]. Passing the current through the reference electrode will drive changes in its composition and subsequently its potential. This will further complicate any voltammetric response obtained and affect any analysis of measured potentials and reaction mechanisms.

These issues associated with the stability of the reference electrode potential are only of concern when measuring the dc potentials of an electrochemical system as described in this manuscript. When measuring the electrophysiological behaviour of tissue, the response is normally high-pass filtered, removing the dc component of the data. As a result, changes in reference electrode potential due to solution composition and electrode polarisation do not affect local field potential and spike data assessed from high frequency electrophysiological recordings.

Electrochemical measurements made in saline use a high conductivity electrolyte, limiting any *iR*_u_ effects. When electrochemistry was performed in vivo, the tissue resistance was significantly higher than in saline. Fitting the EIS produced a solution resistance of several kΩ. The effects of *iR*_u_ become visible in cyclic voltammetry at a level of ~1 mV or more. The voltammetry in this work produced currents up to ~1 μA, so an *R* of 5 kΩ would result in *iR*_u_ = 5 mV. For the implanted electrode voltammetry, regions where the current is large may have a small shift in measured potentials as a result of *iR*_u_. The impact of *iR*_u_ will be greater when the resistance or current is larger. This may occur when using faster voltammetric scan rates, larger electrodes, or the tissue is more resistive (due to larger electrode distances or higher bone content between the electrodes). When *iR*_u_ is present, electrochemical analysis is further complicated, as variations in resistance will also impact its response (i.e., variations in electrode size, distances, and tissue resistivity will affect electrochemical behaviour).

In the current work, the electrode distances were different between animals and over time. As a result, the tissue resistivity will be slightly affected by experimental error. The electrode area also varied along the implant and between each handmade device. This increased the variation across the data set.

The impacts of varying reference electrode potential and *iR*_u_ are not normally assessed or even detected during in vitro measurements. The presence of these effects in vivo can significantly impact the relevance of the in vitro measurements. Appropriate care must be taken to prevent these effects or to measure their impact.

### 4.4. Implications for Controlled Current Stimulation of Tissue

Electrochemical analysis of bionic electrodes is typically confined to voltammetry and EIS; however, they are normally used clinically with short (μs) current controlled (chronopotentiometric) pulses. In general, the charge storage capacity is related to the change in electrode potential during a current pulse [24]. The maximum current can then be limited so the electrode potential does not obtain values which enable water electrolysis. However, it is difficult to assign specific reaction mechanisms to a chronopotentiometric curve. The voltammetry performed in this work indicates that oxygen reduction, platinum oxide reduction and formation, anion adsorption, capacitance, hydrogen adsorption, and water electrolysis can occur during current pulsing. These reactions can have slow kinetics and be irreversible [38]. As a result, a biphasic waveform may be charge balanced, but the reactions on the oxidation and reduction pulses may be different. This can lead to electrode activation over multiple current pulses and a change in the chemical composition of the electrode–tissue interface.

Electrode activation reduced the over-potential for oxygen reduction and platinum oxide reduction and formation. Electrode activation may occur during current pulsing in vivo, so changes in electrode potential would decrease over time. The low oxygen tension in vivo would significantly reduce the charge available from this reaction, so the reduction current would need to be supplied by a different mechanism, and would most likely lead to a more negative reduction potential. The high in vivo resistance will lead to *iR*_u_ effects, with larger currents having a greater impact. Thus, while the electrode potential may overshoot the water electrolysis potential, the true electrode potential may still be safe.

In some commercial devices, electrode performance is assessed by an impedance test [39,40]. This test measures the electrode potential at some point during a current pulse and applies Ohm’s law to calculate a resistance value. An early publication using this technique measured the potential at the end of a current pulse and argued that in vivo changes in response were initially due to fluid build-up, followed by increasing amounts of fibrous tissue growth [40]. Changes in the impedance test have been detected with cell growth over the electrode in vitro and after fibrous tissue formation in vivo, but not with protein fouling in vitro [41,42]. However, these previous reports all perform this impedance test in a different manner, preventing any comparison of results. Measuring a point on a smooth chronopotentiometric curve is prone to significant error, limiting the accuracy of this technique. More fundamentally, Ohm’s law is only applicable to electrical circuits composed of uniform conductors, not electrochemical systems. The results obtained from an impedance test are not equivalent to electrochemical impedance spectroscopy. Values obtained from the impedance test are not real and the sole attribution of changes in response to biological processes is too simplistic. Changes in tissue or solution resistance, bone formation, distance between electrodes, electrode size or roughness, electrode activation, and solution composition could affect the electrode potential during a current pulse and hence the values measured from an impedance test.

### 4.5. Recommended In Vitro Testing Method for Bionic Electrodes

While there has been a tendency to reduce electrochemical information about bionic electrodes to impedance at 1 kHz and a cathodic CSC, this and previous work by our group has shown this type of analysis is too simplistic. Electrochemical reactions are not reducible to Ohm’s law, and require more detailed analyses to understand charge transfer at the electrode–tissue interface.

There is now an effort to prescribe a single testing solution which is valid for all implantable electrodes [43]. This would enable a simple comparison of all stimulation waveforms, new electrode materials, and geometries. However bionic electrodes are used in vastly different regions of the body (e.g., within the brain, in the cochlea, on the retina, within blood vessels, and within muscle). These electrodes may be in contact with different types and concentrations of salt, protein, and other organic species and different cell types. Moreover, a model solution of the brain may have poor relevance to the cochlea.

An electrode material can have specific and non-specific interactions; for instance, proteins will adsorb on most electrodes, partially blocking them [35], while specific anions and amino acids will attach to a platinum electrode but may not interact with other electrode materials [31]. Omitting amino acids which adsorb to platinum from a model solution limits its relevance. Simple solutions, such as PBS [43], are very poor in vivo models for platinum, as the high phosphate concentration adsorbs on the surface, altering the charge transfer mechanisms [21]. Commonly used sulfuric acid solutions used to clean and assess platinum electrodes [44] are also not relevant, as highly polished electrodes are not used clinically; the charge available from hydride and anion adsorption and the protein stability and adsorption in highly acidic solutions are not relevant to the in vivo environment [21]. Moreover, a model solution tailored specifically to platinum may have poor relevance for understanding other materials.

Furthermore, the electrode–tissue interface is dynamic, with changes in composition over time and from electrical stimulation, and protein adsorption is time dependent [3]. Recommending a testing solution composition for reproducible comparisons must also include the method for its use.

We recommend a series of electrochemical testing solutions and experiments for assessing bionic electrodes. These experiments are aimed at understanding the range of behaviours of an electrode, but are limited in their predictive response as the impact of implantation, *iR*_u_, variable reference electrode potential, and local variations in chemical and tissue composition are not captured in an in vitro test.

The electrode should be tested in a simple saline solution (e.g., 0.9%/0.15 M NaCl) which has been degassed with nitrogen or argon for at least 10 min. A second test should then be performed in a degassed artificial interstitial fluid, perilymph, or cerebrospinal fluid to assess the impact of other ions on the electrochemical performance. Different proteins, amino acids, and other organics can then be added to these solutions at typical concentrations and with varying ratios. The electrode should be exposed to the proteins and amino acids for 10 min before testing to ensure reproducible surface adsorption occurs. Finally, electrodes should be tested in degassed foetal bovine serum after 10 min exposure to assess the impact of other chemical species on the electrochemical performance.

Electrochemistry should be performed in a 3-electrode system with a well-defined reference electrode and large counter electrode. Proteins will adsorb on the reference electrode, affecting its potential, so it must be appropriately calibrated. EIS should be performed at 0 V with ~10 mV amplitude over a wide frequency range (e.g., 0.1–100,000 Hz). The entire response should be reported and fit with a simple equivalent circuit. The impedance at low frequencies (e.g., 15 Hz) can be reported. Cyclic voltammetry is used to understand the reaction mechanisms and kinetics of an electrochemical system. The response varies with electrode material, preparation, and solution composition. This prevents the prescription of a single testing protocol. The voltammetric response should be tested over a wide potential range to determine the reactions that occur and their potentials, the changes in response with different model solutions, and after repeated voltammetric cycles. In general, the voltammetric scan rate should be low to minimize *iR*_u_ and aid in translating to any in vivo performance. Current pulsing can be performed, but the response varies significantly with applied waveform and is affected by *iR*_u_, a variable reference electrode, and the local chemical and tissue composition that occurs after implantation [24].

The effective electrode area can be assessed by the addition of a redox mediator such as ruthenium hexamine to the solution at a known concentration and by appropriate fitting of the voltammetric response, depending on the electrode geometry [21,22,37]. The blocking of the electrode by protein adsorption can be assessed by similar methods [35].

## 5. Conclusions

The electrochemical response of platinum electrodes was assessed in vitro and in vivo over 8 weeks. The response depended on solution composition and was affected by electrode polarisation. Initial electrodes showed no electrochemically detectable platinum oxide present. Electrode polarisation oxidised the electrode surface, reducing the over-potential for the reduction of oxygen and platinum oxide. Electrode polarisation also reduced the impedance at low frequencies and increased its admittance. The resistance of the electrochemical circuit increased when the electrode was implanted, with bone having a higher resistance than tissue. Protein fouling and fibrous tissue formation had a minimal impact on the electrochemical properties in comparison to electrode polarisation and implantation in resistive tissue.

When assessing bionic electrode performance, in vitro electrochemical measurements must be performed in representative solutions, including low oxygen concentrations, anions, and organic compositions. Voltammetric response should be undertaken to assess the range of reaction mechanisms that can occur on the electrode. Charge storage capacity and charge density measurements can change significantly after electrode polarisation and subsequently may have limited utility in predicting in vivo performance. The impedance should be measured over a wide frequency range, with low frequencies used to assess electrode function and high frequencies dependant on solution properties. The typical impedance measurement at 1 kHz is a very poor predictor of electrode performance. When performing electrochemical measurements in vivo, the impact of a non-standard reference electrode potential, 2-electrode system, and *iR*_u_ must be taken into account, as they are not typically present during in vitro measurements.

## Figures and Tables

**Table 1:** Percentage area of scala tympani filled by fibrous tissue and bone 48 days–8 weeks after cochlear implantation.

	Ave.	SD
Loose Fibrous Tissue	9.5	8.8
Dense Fibrous Tissue	3.4	1.6
Bone Formation	0.8	0.7

## Supplementary Materials

The following supporting information can be downloaded at: https://www.mdpi.com/article/10.3390/mi13010103/s1, Figure S1. Typical Nyquist plot of a platinum cochlear implant electrode at 0 V with an AC amplitude of 10 mV after storage in saline over 8 weeks. Red—data, black line—fitting with equivalent circuit; Figure S2. Optical micrograph of a stained section of cochlea after 8 weeks implantation. Dotted line defines scala tympani, solid line defines (a) loose fibrous tissue, (b) dense fibrous tissue (c) new bone formation; Table S1. Average, standard deviation and coefficient of variation of impedance (kOhm); Table S2. Electrochemical impedance parameters from equivalent circuit fitting of cochlear implant electrodes.
