# Supplementary material for: Comparison of the In Vitro and In Vivo Electrochemical Performance of Bionic Electrodes
Source: Micromachines (Basel). 2022 Jan 9;13(1):103. doi: 10.3390/mi13010103 (PMC8779563; doi:10.3390/mi13010103)
Supplement: Supplementary file 1 [file micromachines-13-00103-s001.zip › micromachines-1464073-supplementary.pdf]

micromachines

MDPI

Supplementary Material

Comparison of *in vitro* and *in vivo* electrochemical performance of bionic electrodes

Alexander R. Harris[1,2], Carrie Newbold[2,3], Dimitra Stathopoulos[2,3], Paul Carter[4], Robert Cowan[2,3] and Gordon G. Wallace[2,5]

[1] Aikenhead Centre for Medical Discovery, ARC Centre of Excellence for Electromaterials Science, Faculty of Medicine, Dentistry and Health Sciences, University of Melbourne, Melbourne, Vic, 3010, Australia
[2] The HEARing CRC
[3] Department of Audiology & Speech Pathology, 550 Swanston St, University of Melbourne, Melbourne, 3010, Australia
[4] Cochlear Ltd, 1 University Ave, Macquarie University, NSW, 2109, Australia
[5] ARC Centre of Excellence for Electromaterials Science, Intelligent Polymer Research Institute, University of Wollongong, Wollongong, NSW, 2522, Australia
Email: alexrharris@gmail.com

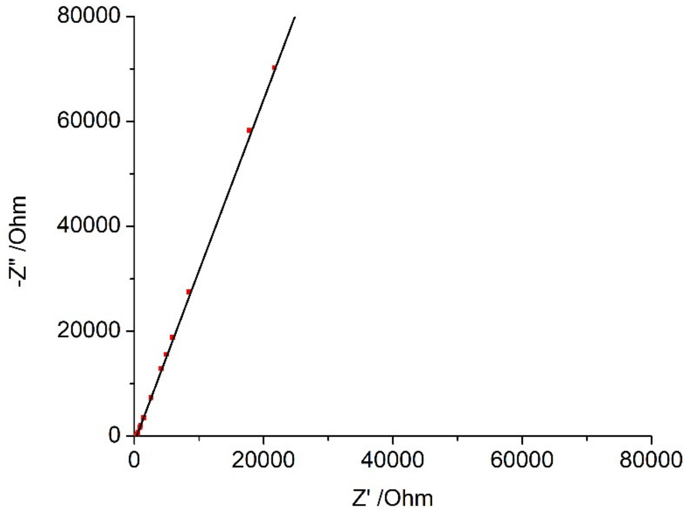

Figure S1. Typical Nyquist plot of a platinum cochlear implant electrode at 0 V with an AC amplitude of 10 mV after storage in saline over 8 weeks. Red – data, black line – fitting with equivalent circuit.

Table S1. Average, standard deviation and coefficient of variation of impedance (kOhm).

**Testing Conditions**			**Impedance 15 Hz**			Impedance 1 kHz		
	**Location**	**Time**	**Ave**	**SD**	**CV**	Ave	SD	CV
Initial	Saline		293	159	0.54	8.1	4.6	0.57
Implanted	Cochlea	1 day	140	65	0.47	6.1	1.9	0.31
Implanted	Subcutaneous	1 day	166	58	0.35	5.6	2.0	0.36
Explanted	Saline	1 day	76	27	0.36	2.6	1.0	0.38
Implanted	Cochlea	4 days	93	31	0.34	4.9	1.0	0.20
Implanted	Cochlea	2 weeks	72	24	0.34	6.5	2.6	0.40
Implanted	Cochlea	8 weeks	111	81	0.73	14.7	5.0	0.34
Implanted	Subcutaneous	4 days	68	30	0.45	3.2	1.2	0.37
Implanted	Subcutaneous	2 weeks	69	36	0.53	3.9	3.0	0.75
Implanted	Subcutaneous	8 weeks	67	9	0.14	4.1	0.2	0.06
Explanted	Saline	4 days - 8 weeks	53	20	0.38	1.8	0.7	0.40
Initial	Saline		431	44	0.10	11.2	1.3	0.11
Stored	Saline	2 weeks	67	16	0.24	2.5	0.7	0.27
Stored	Saline	8 weeks	55	11	0.19	2.0	0.2	0.08
Initial	Saline		215	192	0.89	11.1	10.0	0.91
After 1 Voltammetric Cycle	Saline		47	2	0.04	3.2	0.6	0.18
After 2 Voltammetric Cycles	Saline		41	1	0.02	2.8	0.4	0.15
After 3 Voltammetric Cycles	Saline		37	2	0.06	2.8	0.4	0.15

Table S2. Electrochemical impedance parameters from equivalent circuit fitting of cochlear implant electrodes.

**Testing Conditions**			**Solution Resistance /Ohm**			**Q_0 / 10^{-9} S $s^{1/2}$**			*n*	
	**Location**	**Time**	**Ave**	**SD**	**CV**	**Ave**	SD	CV	Ave	SD
Initial	Saline		322	54	0.16	112	123	1.10	0.85	0.03
Implanted	Cochlea	1 day	2190	455	0.21	1350	7190	5.31	0.78	0.07
Implanted	Subcutaneous	1 day	1330	333	0.25	218	193	0.88	0.80	0.03
Explanted	Saline	1 day	353	74	0.21	421	288	0.68	0.81	0.04
Implanted	Cochlea	4 days	2630	655	0.25	381	191	0.50	0.78	0.04
Implanted	Cochlea	2 weeks	3860	1580	0.41	606	383	0.63	0.74	0.08
Implanted	Cochlea	8 weeks	3720	2310	0.62	1430	702	0.49	0.50	0.08
Implanted	Subcutaneous	4 days	1180	300	0.25	554	270	0.49	0.77	0.01
Implanted	Subcutaneous	2 weeks	1430	780	0.55	695	319	0.46	0.75	0.09
Implanted	Subcutaneous	8 weeks	1410	330	0.3	623	98	0.16	0.71	0.00
Explanted	Saline	4 days - 8 weeks	354	68	0.19	607	390	0.64	0.81	0.03
Initial	Saline		364	21	0.06	46	6	0.14	0.87	0.01
Stored	Saline	2 weeks	357	108	0.30	434	116	0.27	0.79	0.01
Stored	Saline	8 weeks	341	23	0.07	513	109	0.21	0.80	0.02
Initial	Saline		710	143	0.20	286	245	0.86	0.72	0.01
After 1 Voltammetric Cycle	Saline		702	12	0.02	990	125	0.13	0.68	0.04
After 2 Voltammetric Cycles	Saline		679	16	0.02	1260	143	0.11	0.66	0.05
After 3 Voltammetric Cycles	Saline		673	11	0.02	1330	79	0.06	0.66	0.03

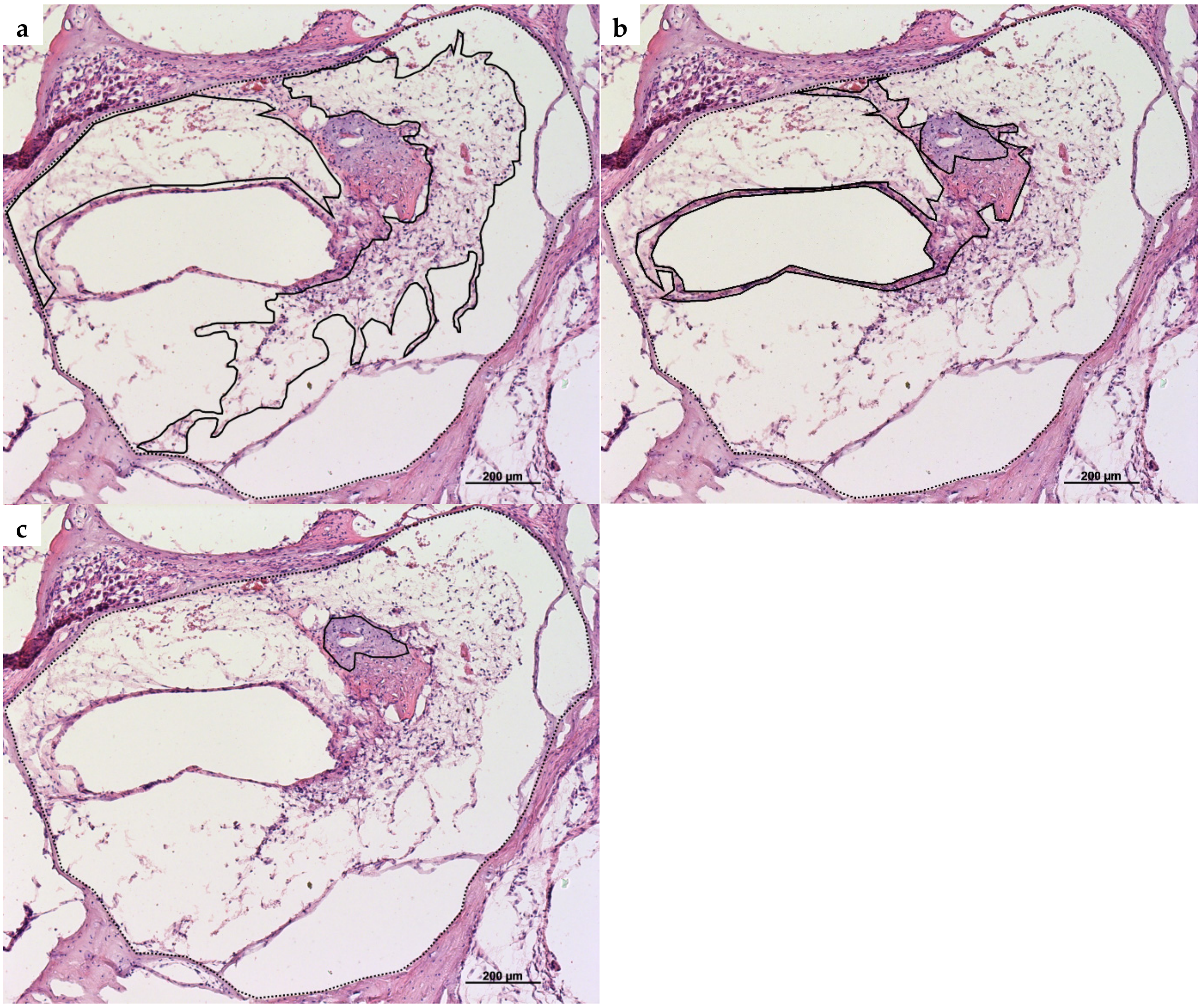

Figure S2. Optical micrograph of a stained section of cochlea after 8 weeks implantation. Dotted line defines scala tympani, solid line defines (a) loose fibrous tissue, (b) dense fibrous tissue (c) new bone formation.
